# The Role of Hypofractionated Radiotherapy in Prostate Cancer

**DOI:** 10.1007/s11912-017-0584-7

**Published:** 2017-03-25

**Authors:** Linus C. Benjamin, Alison C. Tree, David P. Dearnaley

**Affiliations:** 1 Division of Radiotherapy and Imaging, The Institute of Cancer Research and The Royal Marsden Hospital NHS Foundation Trust, Downs Road, Sutton Surrey, SM2 5PT London, UK; 2 Royal Marsden NHS Foundation Trust and the Institute of Cancer Research, Downs Road, Sutton Surrey, SM2 5PT London, UK

**Keywords:** Prostate cancer, Radiotherapy, Hypofractionation, CHHiP, HYPRO

## Abstract

**Purpose of Review:**

It is now accepted that prostate cancer has a low alpha/beta ratio, establishing a strong basis for hypofractionation of prostate radiotherapy. This review focuses on the rationale for hypofractionation and presents the evidence base for establishing moderate hypofractionation for localised disease as the new standard of care. The emerging evidence for extreme hypofractionation in managing localized and oligometastatic prostate cancer is reviewed.

**Recent Findings:**

The 5-year efficacy and toxicity outcomes from four phase III studies have been published within the last 12 months. These studies randomizing over 6000 patients to conventional fractionation (1.8–2.0 Gy per fraction) or moderate hypofractionation (3.0–3.4 Gy per fraction). They demonstrate hypofractionation to be non-inferior to conventional fractionation.

**Summary:**

Moderate hypofractionation for localized prostate cancer is safe and effective. There is a growing body of evidence in support of extreme hypofractionation for localized prostate cancer. Extreme hypofractionation may have a role in managing prostate oligometastases, but further studies are needed.

## Introduction

Prostate cancer (PCa) is the second most commonly diagnosed male malignancy [1] in the Western world, with the majority of patients having organ-confined disease at presentation [2].

Radical or curative radiotherapy has been traditionally given with a conventionally fractionated schedule, using daily dose of 1.8–2.0 Gy over 7–8 weeks, to a total dose of 74–79.2 Gy.

The dose of 1.8–2.0 Gy per fraction in conventional fractionation (CF) is based on the presumed relative sensitivity of malignant and normal tissue. The radiobiology and response of tumours and normal tissue to total dose and dose per fraction has been the subject of intense research for more than 30 years [3, 4]. These studies led to the development of the linear-quadratic model, which describes the relationship between cell survival, dose and dose per fraction [5]. In the linear-quadratic model, the response of tissue to fraction size is described by the alpha/beta ratio (α/β). The α/β for most tumours is >8 Gy, while that for late-responding normal tissue is estimated at 3–4 Gy. For these tumours, CF at 1.8–2.0 Gy per fraction results in an improvement in the therapeutic ratio.

In parallel advances in physics, engineering, computing and imaging have been channelled into the development of image-guided intensity-modulated radiotherapy. The improvements in imaging give better target definition, and it is now possible to accurately deliver highly conformal treatment. This has made it possible to both reduce radiation-related side-effects and escalate dose. Several phase III studies in prostate cancer have shown that increasing dose, improves biochemical disease-free survival, with acceptable acute and long-term toxicities [6–9]. If CF is used, dose-escalated radiotherapy is now the standard of care.

Dose escalation has been achieved through and increase in the number of fractions delivered. This has resulted in prolongation of total treatment time, with an increased number of hospital visits for patients, as well as greater departmental workload and higher costs. With an improvement in the understanding of the radiobiology of PCa, hypofractionation has become an attractive means of dose escalation, without prolonging treatment duration.

## Rationale for Hypofractionation

The theoretical basis for hypofractionation in PCa is the comparatively low α/β for PCa. Evidence supporting the hypothesis for a very low α/β for PCa has become available in the last two decades, including pre-clinical and clinical data. In 1999, a study of 367 patients calculated an α/β of 1.5 Gy (95% CI 0.8–2.2) [10]. A subsequent study [11] with 1020 patients treated with external beam radiotherapy and brachytherapy derived the same value (1.5; 95% CI 1.25–1.75). These early findings have been supported in a retrospective analysis of 6000 patients treated with external beam radiotherapy [12], which calculated an α/β of 1.4 (95% CI 0.9–2.2), with no significant difference in the calculated ratio between different risk groups or with androgen deprivation. In conjunction with other studies [13–15], the α/β of PCa can be estimated at 1.4–1.9 Gy. This estimate does not take into account the potential effects of treatment duration or accelerated repopulation [14].

The low α/β estimates for PCa suggest a greater sensitivity to increasing fraction size, raising the possibility of dose-escalation through hypofractionation. The α/β for dose-limiting organs in prostate radiotherapy is postulated to be comparatively higher (rectum and bladder; α/β 3–5 Gy). This forms the theoretical basis for an improvement in the therapeutic ratio of radiotherapy with larger fraction sizes, while delivering an isoeffective dose to the prostate.

## Moderate Hypofractionation

Moderate hypofractionation (MH) refers to the delivery of 2.4–4.0 Gy per fraction, daily, over 4–6 weeks.

Two systematic reviews [16, 17] of the randomized evidence for prostate MH had previously concluded that there was insufficient evidence to demonstrate that MH produces improved outcome compared to CF. However, new data from four randomized studies published within the last 12 months [18••, 19–21, 22•, 23•, 24•] are now available. Collectively, 6357 patients have been randomized to CF (1.8–2.0 Gy per fraction) or MH (3.0–3.4 Gy per fraction), and 5-year efficacy outcomes and toxicity profiles have been reported. These results show that MH is as well tolerated and as clinically effective as CF, with the obvious economic and practical advantages associated with shorter treatment durations.

### Moderate Hypofractionation for Localized Disease

Randomized data comparing MH to CF includes two early trials, four modern superiority trials, and three modern non-inferiority trials (Table 1).

**Table 1:** Summary table of randomized studies comparing moderate hypofractionation and conventional fractionation in prostate cancer

Study	Risk	Technique	ADT (%)	Number of patients	Fractionation total dose/fractions/dose per fraction	Treatment duration	BED α/β 1.8 Gy	BED α/β 3.0 Gy	BED α/β 10 Gy	Acute toxicity ≥grade 2 GI	Acute toxicity ≥grade 2 GU	Late toxicity ≥grade 2 GI	Late toxicity ≥grade 2 GU	5-year biochemical relapse-free survival (%)
Superiority randomized studies														
Arcangeli [30]	LR/IR 24% HR 76%	CFRT	100	85	80 Gy/40 fractions/2 Gy per fraction	8 weeks	168.9	133.3	96.0	21	40	14	11	92.0
			100	83	62 Gy/20 fractions/3.1 Gy per fraction	4 weeks	168.8	126.1	81.2	35	47	17	16	96.0
Hoffman [29]	LR 28% IR 71% HR 1%	IG-IMRT	23	101	75.6 Gy/42 fractions/1.8 Gy per fraction	8.4 weeks	151.2	121.0	89.2			5.1	16.5	92.0
			25	102	72 Gy/30 fractions/2.4 Gy per fraction	6 weeks	168.0	129.6	89.3			10	15.8	96.0
Pollack [31]	IR 36% HR 64%	IMRT	47	151	76 Gy/38 fractions/2 Gy per fraction	7.6 weeks	160.4	126.7	91.2		47.7	22.5	13.4	85.0
			45	152	70.2 Gy/26 fractions/2.7 Gy per fraction	5.2 weeks	175.5	133.4	89.2		44.9	18.1	21.5	81.0
HYPRO [2–17, 18••, 19–21, 22•]	IR 27% HR 73%	CFRT	67	410	78 Gy/39 fractions/2 Gy per fraction	7.8 weeks	164.7	130.0	93.6	31.2	57.8	17.7 (G3 + toxicity 2.6%)	39 (G3 + toxicity 12.9%)	77.0
			67	410	64.6 Gy/19 fractions/3.4 Gy per fraction	6.5 weeks	186.6	137.8	86.6	42	60.5	21.9 (G3 + toxicity 3.3%)	41.3 (G3 + toxicity 19%)	81.0
Non-inferiority randomized studies														
RTOG 0415 [23•]	LR	IMRT 79–80% CFRT 20–21%	0	542	73.8 Gy/41 fractions/1.8 Gy per fraction	8.2 weeks	147.6	118.1	87.1	10.3	27.1	14	22.8	85.3
			0	550	70 Gy/28 fractions/2.5 Gy per fraction	5.6 weeks	167.2	128.3	87.5	10.7	27	22.4	29.7	86.3
PROFIT [24•]	IR	IGRT	0	598	78 Gy/39 fractions/2 Gy per fraction	7.8 weeks	164.7	130.0	93.6			>G3 GI/GU 5.4%		79.0
		IGRT	0	608	60 Gy/20 fractions/3 Gy per fraction	4 weeks	160.0	120.0	78.0			>G3 GI/GU 3.5%		79.0
CHHiP [18••, 19, 27]	LR 15% IR 73% HR 12%	IMRT +/− IGRT	97	1065	74 Gy/37 fractions/2 Gy per fraction	7.4 weeks	156.2	123.3	88.8	25	46	13.7	9.2	88.3
			97	1074	60 Gy/20 fractions/3 Gy per fraction	4 weeks	160.0	120.0	78.0	38	49	12	11.7	90.6
			97	1077	57 Gy/19 fractions/3 Gy per fraction	3.8 weeks	152.0	114.0	74.1	38	46	11.2	6.6	85.9

*LR/IR/HR* low/intermediate/high risk prostate cancer, *CFRT* conformal radiotherapy, *IMRT* intensity-modulated radiotherapy, *IGRT* image-guided radiotherapy, *BED* biological effective dose, *ADT* androgen deprivation therapy, *GI* gastrointestinal, *GU* genitourinary

#### Early Studies

The earliest randomized studies in MH were undertaken in Canada [25] and Australia [26] and gave conflicting results. Their design was motivated by logistical benefits of shorter treatment durations, in countries where patients had to travel considerable distances for radiotherapy. The doses in the arms these early studies were not isoeffective, as no assumptions about the α/β of PCa were made in the design of the trials.

The Canadian trial [25] enrolled 936 patients with low- or intermediate-risk disease, randomizing them to 66 Gy/33 fractions/6.6 weeks or 52.5 Gy/20 fractions/4 weeks. The biologically effective dose (BED) in the hypofractionated arm was lower than that in the standard arm. The study reported higher rates of 5-year biochemical failure (60 vs 53%; *p* < 0.05) and acute grade 3/4 gastrointestinal (GI) and genitourinary (GU) toxicity (11 vs 7%) in the hypofractionated arm.

However, after a median follow-up of 5.7 years, there was no difference in late GI/GU toxicity (≥grade 3 late toxicity 3.2%) between the two study arms.

The Australian trial [26] randomized 217 patients, with favourable risk PCa, to 64 Gy/32 fractions/6.4 weeks or 55 Gy/20 fractions/4 weeks. Treatment was delivered using a 2D, four-field box, technique.

After a median follow-up of 90 months, biochemical disease-free survival was significantly better for the hypofractionated arm (53 vs 34%; *p* < 0.05), with no significant difference in GI/GU toxicity or overall survival. Multivariate analysis revealed CF to be an independent predictor for biochemical failure and GU toxicity at 4 years.

While these early studies demonstrated the feasibility of MH, their toxicity and efficacy outcomes are not applicable in modern radiotherapy, as the radiotherapy techniques and doses employed in these studies are no longer in keeping with the current standard of care.

#### Modern Studies

The modern MH trials assume that the α/β for PCa is 1.5 Gy, and have complementary design, addressing different hypotheses. The superiority studies hypothesize a greater efficacy of hypofractionation with equivalent toxicity, while the non-inferiority studies aim to demonstrate equivalent efficacy with reduced or similar toxicity.

There are three large randomized non-inferiority trials (CHHiP [18••, 19, 27], RTOG 0415 [23•] and PROFIT [24•]) evaluating the equivalence of MH and CF. The doses in the MH arms in these studies range from 57 to 70 Gy in 2.5–3.4 Gy per fraction. Overall, these studies demonstrate that the safety and efficacy of MH is similar to that of CF.

The largest non-inferiority randomized study of MH is the CHHiP study [18••, 19, 27]. This study enrolled 3216 patients from 71 centres in the UK, Ireland, Switzerland and New Zealand. Patients were randomized to 74 Gy/37 fractions/7.4 weeks, 60 Gy/20 fractions/4 weeks, or 57 Gy/19 fractions/3.8 weeks, with treatment delivery using IMRT. The experimental fractionation schedule were designed to be isoeffective for α/β of 2.5 Gy (60-Gy schedule) and 1.5 Gy (57-Gy schedule). Twelve, 73, and 15% of patients in this study had low-, intermediate- or high-risk disease, respectively. Short-course hormonal therapy was mandated for patients with intermediate- or high-risk disease.

The primary end point in the CHHiP study was time to biochemical failure, with the critical hazard ratio for non-inferiority being 1.208.

After a median follow-up of 62.4 months, the 5-year biochemical or clinical failure-free survival was found to be 88.3% in the 74-Gy arm (95% CI 86.0–90.2), 90.6% in the 60-Gy arm (95% CI 88.5–92.3), and 85.9% in the 57-Gy arm (95% CI 83.4–88.0). The 60-Gy arm was non-inferior to the 74 Gy (HR 0.84, 90% CI 0.68–1.03; pNI = 0.0018). The 57-Gy arm was not non-inferior to the 74-Gy arm (HR 1.20, 90% CI 0.99–1.46). Overall mortality in each arm was similar; 8.6, 6.8 and 8.1% in the 74, 60 and 57-Gy arm, respectively. There were no statistically significant differences between the arms with respect to distant metastasis rate (3.0, 2.7 and 3.9% for the 74, 60 and 57Gy arms, respectively).

While acute RTOG GI/GU toxicity had become similar in each arm by 18 weeks, it peaked earlier in the hypofractionated arm (4–5 weeks) compared to the control arm (7–8 weeks). Early GI ≥grade 2 toxicity was significantly higher in the hypofractionated arms; it was 25% in the 74-Gy arm, 38% in the 60-Gy arm (*p* < 0.0001) and 38% in the 57-Gy arm (*p* < 0.0001).

5-year clinician and patient-reported side-effects were not significantly different. RTOG grade ≥2 GI toxicity was reported at 13.7, 11.9 and 11.5% in the 74-, 60- and 57-Gy arms respectively. Grade ≥2 GU toxicity was reported at 9.1, 11.7 and 6.6% in the 74-, 60- and 57-Gy arms, respectively.

Comparison of the 60- and 57-Gy arms revealed a slightly higher rate of cumulative LENT-SOM grade ≥2 GI toxicity (HR 1.39, 95% CI 1.14–1.70; *p* = 0.001) and GU toxicity (HR 1.58, 95% CI 1.13–2.20; *p* = 0.007).

The CHHiP [18••, 19, 27] study provides compelling evidence for hypofractionation, with the authors recommending 60 Gy/20 fractions/4 weeks becoming the new standard of care for the management of localized PCa.

The PROFIT study (NCT00304759) has recently been presented [24•]. The critical hazard ratio for non-inferiority in this study was set at 1.32. PROFIT [24•] recruited 1206 men with intermediate-risk disease, randomizing them to 60 Gy/20 fractions/4 weeks or 78 Gy/39 fractions/7.8 weeks. All patients in PROFIT [24•] had intermediate-risk disease, with none receiving hormonal therapy.

After a median follow-up of 6 years, no significant difference in 5-year biochemical failure (HR 0.96, 90% CI 0.80–1.15), acute ≥grade 3 GI/GU toxicity, or overall survival have been reported. Interestingly late toxicity was lower in the MH arm (3.5 vs 5.4%, difference = −1.9%, 95% CI −4.3 to 0.43).

The authors of PROFIT [24•] conclude that, for patients with intermediate-risk disease, MH is non-inferior to CF, for both efficacy and acute/late toxicity.

The RTOG 0415 [23•] study randomized 1092 patients, with low-risk disease, to 73.8 Gy/41 fractions/8.2 weeks or 70 Gy/28 fractions/5.6 weeks. The critical hazard ratios for non-inferiority were set at 1.52 for 5-year disease-free survival (primary end point), 1.67 for cumulative biochemical recurrence and 1.54 for overall survival.

After a median follow-up of 5.8 years, the MH arm was reported to be non-inferior to the CF arm with respect to 5-year disease-free survival (HR 0.85, 95% CI 0.64–1.14; *p* < 0.001), biochemical recurrence (HR 0.77, 95% CI 0.51–1.17; *p* < 0.001) and overall survival (HR 0.95, 95% CI 0.64–1.41; *p* = 0.008).

The acute side effects did not differ significantly in the two arms of the study. The MH arm had a significantly higher rate of grade 2–3 late GI toxicity (22.4 vs 14%; RR 1.55–1.59) and grade 2–3 late GU toxicity (29.7 vs 22.8%; RR 1.31–1.59).

The authors concluded that the efficacy of MH is not inferior to CF, though the late grade 2–3 GI/GU toxicity is higher.

CHHiP [18••, 19, 27], RTOG 0415 [23•] and PROFIT [24•] include patients in different risk groups and differ in the use of hormonal therapy, but all studies give very similar hazard ratios (<1.0) for their primary end points, demonstrating that the efficacy of MH is not inferior to CF. They differ in their late toxicity outcomes.

In contrast to RTOG 0415 [23•],CHHiP [18••, 19, 27] has reported no difference in late toxicity, while PROFIT [24•] reports a lower rate of late toxicity in the hypofractionated arm. These differences may partly be accounted for by the BED in the hypofractionated and control arms of each study. Assuming an α/β of 3.0 Gy for bladder/rectum, the BED in the hypofractionated arm is higher than the control arm in RTOG 0415 (128 Gy vs 118 Gy), similar to the control arm in CHHiP [18••, 19, 27] (120 Gy vs 123 Gy), and lower than the control arm in PROFIT [24•] (120 Gy vs 130 Gy).

Comparing PROFIT [24•] and CHHiP [18••, 19, 27], the use of hormonal therapy in some patients in CHHiP appears to improve biochemical control by 10%, although the impact of hypofractionation is similar with or without hormonal therapy.

Finally, the α/β estimated by CHHiP [18••, 19, 27] (1.8 Gy) and PROFIT [24•] (1.3 Gy) are both in keeping with the low range of 1.4–1.9 Gy estimated from meta-analyses and large series [12–15], further re-enforcing the theoretical basis for MH.

In the four modern MH superiority randomized trials [20, 21, 22•, 28–31], the dose in the MH arms ranges from 62 to 72 Gy in 2.4–3.4 Gy per fraction. Collectively, these studies have not demonstrated any differences in efficacy after 5 years. No differences in metastasis-free, cancer-specific survival or overall survival have been demonstrated.

Hoffman [28, 29] and Pollack [31] compared CF with MH and found no significant difference in 5-year biochemical recurrence-free survival.

Arcangeli [30] recruited 168 patients with high-risk PCa, randomizing them to 80 Gy/40 fractions/8 weeks or 62 Gy/20 fractions/4 weeks, in conjunction with 9 months of hormonal therapy.

After a median follow-up of 70 months, a non-significant improvement in actuarial 5-year biochemical recurrence-free survival was demonstrated in the MH arm (85 vs 79%; *p* = 0.065). No significant difference in local or distant recurrence was demonstrated. However, subgroup analysis of patients with a PSA ≤20 ng/ml revealed a significant improvement in 5-year local and distant disease control, in addition to biochemical control.

The HYPRO study [20, 21, 22•] is the largest of the MH superiority studies. HYPRO randomized 804 patients, with intermediate- or high-risk disease to 64.6 Gy/19 fractions/3 fractions per week/6.5 weeks or 78 Gy/39 fractions/5 fractions per week/7.8 weeks. The majority of patients in this study were high-risk (>70%), with 66% receiving concomitant hormonal therapy. The primary end point was 5-year relapse-free survival. An additional, non-inferiority, end point was the incidence of ≥grade 2 GI/GU toxicity, with a critical hazard ratio designated as 1.11/1.13, respectively.

After a median follow-up of 60 months, the 5-year relapse-free survival was not statistically different in the MH and CF arms (77.1 vs 80.5%; *p* = 0.36).

No differences in acute ≥grade 2 GU toxicity were reported between the MH (60.5%, 95% CI 55.8–65.3) and CF arms (57.8%, 95% CI 52.9–62.7%). However, the cumulative incidence for acute ≥grade 2 GI toxicity was significantly higher (OR 1.6; *p* = 0.0015) in the MH arm (42%, 95% CI 37.2–46.9%) compared to control (31.2%, 95% CI 26.6–35.8%). Furthermore, the cumulative incidence of late ≥grade 3 GU toxicity was significantly higher in the MH arm (19 vs 12%; *p* = 0.021). No statistically significant difference in cumulative ≥ grade 3 late gastrointestinal toxicity was found the two study arms (2.6 vs 3.3%).

In contrast to CHHiP [18••, 19, 27] and PROFIT [24•], HYPRO [20, 21, 22•] concludes that hypofractionation is not non-inferior to CF, with respect to ≥grade 3 late GU toxicity.

For similar reasons to the higher toxicity seen in RTOG 0415 [23•], the higher late toxicity in HYPRO [20, 21, 22•] may relate to a higher BED delivered to organs at risk in the hypofractionated arm of HYPRO.

Additionally, compared to CHHiP [18••, 19, 27], HYPRO [20, 21, 22•] included a greater proportion of the seminal vesicles in the high-dose volume, which may account for the higher late toxicity. In HYPRO, patients with >10% risk of seminal vesicle involvement had their seminal vesicle included in the high-dose volume. Patients with a 10–25% probability of seminal vesicle involvement received a total dose of 70–72.15 Gy/1.85–2.0 Gy per fraction in the control arm or 54.4–57.76 Gy/3.04–3.4 Gy per fraction in the hypofractionated arm. For patients with a >25% risk of seminal vesicle involvement, the seminal vesicles were treated to the full dose of 78 Gy or 64.6 Gy in each respective arm. By contrast, in CHHiP [18••, 19, 27], patients with >15% risk of seminal vesicle involvement received 96% of the prescribed dose to the base of the seminal vesicles, and 80% of the prescribed dose to the seminal vesicles, within each arm.

The outcome of HYPRO [20, 21, 22•] is in keeping with other superiority hypofractionation trials, which did not demonstrate an improvement in efficacy outcomes with hypofractionation. Assuming α/β ratio of 1.5 Gy for PCa, the 2 Gy equivalent dose in the hypofractionated arm of HYPRO is 90.4 Gy. This dose escalation, over the control arm of 78 Gy/39 fractions, may have been expected to have resulted in a significant improvement in biochemical control. Its failure to do so may relate to the longer duration over which treatment was delivered (6.5 weeks), compared to other hypofractionation schedules.

## Extreme Hypofractionation

**Fig. 1:**
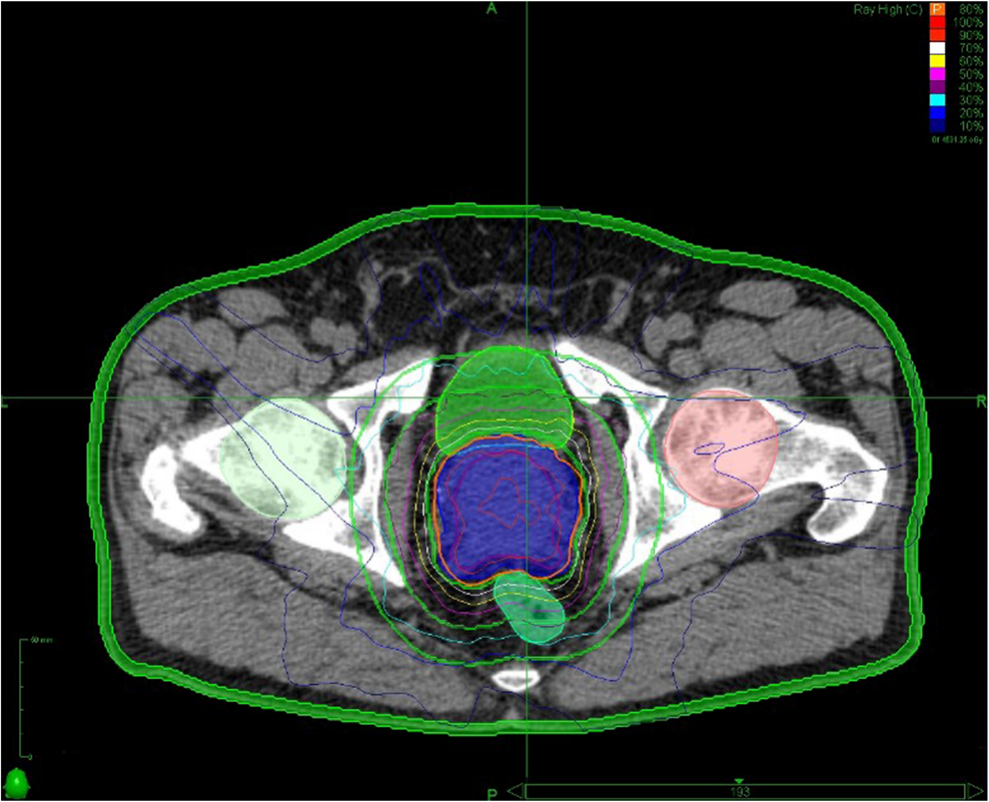
Treatment plan and dose statistics for prostate SBRT with a non-coplanar technique. (Acknowledgement: Dr Nicholas van As, Kirsty Morrison, Royal Marsden Hospital, UK)

Extreme hypofractionation (EH) using stereotactic body radiotherapy (SBRT) refers to the delivery of 6–10 Gy per fraction either daily, on alternate days or weekly, to a total dose of 35–50 Gy (Fig. 1). While the linear-quadratic model predicts an improvement in therapeutic ratio with MH, its applicability to EH has been questioned [32], because it does not account for vascular and stromal tissue injury occurring at EH. However, this criticism relates to doses above 10 Gy per fraction, which are not used in EH of PCa radiotherapy [33]. At doses below 10 Gy per fraction, the linear-quadratic model seems to predict tumour control well, without the need for additional factors [34].

### Extreme Hypofractionation for Localized Disease

Two systematic reviews [16, 17] have recommended that EH be only pursued in the setting of a clinical trial, owing to the absence of long-term data. In the last decade, phase I–II data using either IMRT [35–38] or non-coplanar techniques [39–45] have demonstrated toxicity and efficacy outcomes of EH being comparable to CF. Patient-reported quality-of-life outcomes are similar between IMRT, SBRT and LDR brachytherapy at 2 years [46]. SBRT compares favourably with IMRT and LDR brachytherapy in terms physician-reported toxicity outcomes [47].

Outcomes with prostate SBRT have been encouraging for doses between 33.5 and 40 Gy/6.7–8.0 Gy per fraction. Based on a study by Kim [38], doses > 9Gy per fraction to a total of >45Gy to the whole prostate are not recommended, owing to higher risk of toxicity. In this dose-escalation SBRT study, patients with low-intermediate-risk disease, received 45 Gy/5 fractions/9 Gy per fraction, 47.5 Gy/5 fractions/9.5 Gy per fraction, or 50 Gy/5 fractions/10 Gy per fraction. After a median follow-up of 24 months, 6.6% of patients treated with 50 Gy/5 fractions had ≥grade 3 late rectal toxicity.

Studies treating patients to 33.5–36.25 Gy/5 fractions/6.7–7.25 Gy per fraction have reported good early outcomes with acceptable toxicities.

Madsen [35] treated 40 patients to a dose of 33.5 Gy/5 daily fractions/6.7 Gy per fraction. After a median follow-up of 41 months, biochemical disease-free survival was 90%, with no incidence of ≥ grade 3 GI/GU toxicity. Grade 2 GU/GI toxicity was reported at 20 and 8%, respectively.

Loblaw [36] treated 84 patients with low-risk disease, with 35 Gy/5 weekly fractions/7 Gy per fraction. After a median follow-up of 55 months, biochemical relapse-free survival was reported at 98%. No acute GU toxicity ≥grade 3 was reported. Late grade 2 GU/GI toxicity was 7 and 5%, respectively.

Chen [41] treated 100 low-high-risk patients with 36.25 Gy/5 alternate day fractions/7.25 Gy per fraction. After a median follow-up of 27 months, the biochemical disease-free survival was 99%, with no acute ≥grade 3 GI/GU toxicity. The late ≥grade 3 GU toxicity rate was 1%. Grade 2 acute and chronic GU toxicity was 35 and 31%, respectively (CTCAE); this is higher than that reported in other studies [40, 44], primarily as it relates to the use of α-antagonists in the study. The higher reported toxicity, did not have a significant impact on patients’ long-term quality of life [49]. Acute and late GI toxicity was 5 and 1%, respectively.

The above studies measured acute toxicity only once in the first 3 months after SBRT. Studies that have evaluated acute toxicity more than once in the first 3 months after SBRT [42, 50] have reported higher toxicity rates.

Bolzicco [42] treated 100 patients with low-high-risk disease, with 35 Gy/5 daily fractions/7 Gy per fraction. No acute grade 3 toxicity was reported. Acute GI/GU toxicity was 18 and 12%, respectively. Late GI/GU toxicity was 1 and 3%, respectively. The biochemical relapse-free survival was 95% after a median follow-up of 36 months.

Tree [50] has reported on a series of 51 patients treated with 36.25 Gy/5 alternate day fractions/7.25 Gy per fraction. Acute grade 2 GU/GI toxicity was 22 and 14%, respectively, with 4% of patients having acute grade 3 urinary toxicity.

Long-term outcomes for prostate SBRT have been reported by Meier [45] and King [48].

A multi-institutional study [45] recruited 309 patients, with low-intermediate-risk disease, treating the prostate to 40 Gy/5 fractions/8 Gy per fraction and the seminal vesicles to 36.25 Gy/5 fractions/7.25 Gy per fraction. After a median follow-up of 61 months, 1.6% of patients reported grade 3 toxicity, all of which were GU toxicities. No grade 4–5 toxicity was reported. The 5-year overall survival and biochemical disease-free survival rates were 95.6 and 97.1% respectively.

King performed a pooled analysis of 1100 patients, treated with SBRT [48]. Eleven percent of patients had high-risk disease. At a median follow-up of 36 months, the 5-year biochemical disease-free survival for low-, intermediate- and high-risk disease was 95, 84, and 81%, respectively.

While the phase I–II data on prostate SBRT is encouraging, the phase III data is eagerly awaited. There are two phase III studies comparing EH with CF.

The Swedish HYPO trial (ISRCTN45905321) has randomized 592 patients, with intermediate-risk disease to CF or 42.7 Gy/7 alternate day fractions/6.1 Gy per fraction.

The PACE B study (NCT01584258) is ongoing, and randomizes patients to CF or 36.25 Gy/5 fractions/7.25 Gy per fraction/5 days. In view of the recently published CHHiP study [18••], a hypofractionated protocol amendment has been developed for PACE B, allowing 62 Gy/20 fractions/3.1 Gy per fraction/4 weeks.

The PATRIOT study is evaluating alternate day (treatment duration 11 days) versus weekly (treatment duration 29 days) stereotactic prostate radiotherapy. This study, delivers 40 Gy/5 fractions/8 Gy per fraction to the prostate and evaluates bowel quality-of-life parameters. Early results suggest superior quality of life with respect to bowel and urinary function, in the first 3 months following treatment, in the 29-day arm of the study [51].

### Extreme Hypofractionation in Oligometastatic Disease

Oligometastatic PCa, referring to ≤3 isolated sites of metastatic disease, is increasingly being diagnosed, partly because of the development of sophisticated imaging techniques. Historically, the treatment of oligometastatic disease has been identical to that of polymetastatic disease; however, oligometastatic disease may represent a biologically distinct clinical state [52]. Aggressive treatment of oligometastases may help eliminate castrate-resistant clones, delay the development of castrate-resistant disease and thereby potentially improve survival [53].

The data on EH for oligometastatic disease is limited. The two largest studies of SBRT in oligometastatic disease each had 50 patients with predominantly nodal or osseous disease [54, 55].

Decaestecker [54] treated metastases to 50 Gy/10 fractions with 1 month of hormonal therapy or 30 Gy/3 fractions without hormonal therapy. The median progression-free survival was 19 months, and a 2-year progression-free survival of 35%. Grade 2 toxicity was reported in 6% of patients.

Schick [55] reported 3-year progression-free survival of 59%. In comparison to the Decaestecker study, the Schick study treated a greater proportion of patients (98 vs 70%) with longer duration of hormonal therapy (1 year vs 1 month). Additionally, in those patients treated for oligometastic nodal disease, more patients received prophylactic irradiation in the Schick study (61 vs 0%).

Jereczek-Fossa [56] treated 19 patients with predominantly pelvic nodal oligometastatic disease to a dose of 33–36 Gy/3 fractions; all patients were receiving between 12 and 17 months of hormonal therapy. The 30-month progression-free survival was 63.5%.

Casamassima [57] treated 25 patients with nodal oligometastatic disease, to 30 Gy/3 fractions, without hormonal therapy. The median progression-free survival was 24 months, with no ≥grade 2 toxicity.

Muacevic [58] reported on 40 patients with mainly spinal oligometastatic osseous disease. An actuarial 2-year local tumour control rate, as measured by MRI and PET-CT was reported at 95.5%.

A systematic review of retrospective studies [59] reported on 299 patients with oligometastatic disease treated with radiotherapy, 55% of whom were treated with extreme hypofractionation. Of the data available for acute toxicity, grade 1–2 toxicity was reported in 15% of patients, with grade 3 toxicity in 0.7%.

Pooled analysis of 119 patients, with ≤3 metastases, treated with SBRT has been reported by Ost [60, 61]. The local control was found to be superior when SBRT delivered a BED ≥100 Gy. No ≥grade 3 toxicities were reported, with 3% of patients reporting grade 2 toxicity. The 3-year distant progression-free survival was reported at 31%.

Pooled analysis [60] of a subset of 72 patients with oligorecurrent nodal disease, treated with SBRT at a dose of 5Gy per fraction to a BED of at least 80Gy, has reported a median distant progression-free survival of 21 months, with a progression-free survival of 34%/13% at 3/5 years, respectively. Late grade 1 and 2 toxicity was reported in 17 and 4% of patients, respectively.

From these studies, SBRT appears to achieve local control of oligometastatic disease; however, randomized studies are needed to establish its impact on progression-free survival and overall survival.

Several prospective studies are currently underway evaluating SBRT in oligometastatic disease (the phase II CORE study NCT02759783, the SABR-COMET study NCT01446744, the STOMP study NCT01558427, the ORIOLE study NCT02680587).

## Conclusions

Moderate hypofractionation for localized PCa is safe and effective. Based on several large randomized studies, hypofractionation at around 3 Gy per fraction can be considered a standard of care for localized PCa.

There is a growing body of evidence in support of extreme hypofractionation for localized PCa. Comparative data and the data from several randomized studies are awaited. Prostate oligometastases represent another scenario where extreme hypofractionation may have a role but the results of ongoing studies are needed before this is considered the standard of care.
